# The effect of background information and motion speed on the performance of TTC estimation

**DOI:** 10.1186/s40359-023-01502-x

**Published:** 2024-01-05

**Authors:** Yao Tong, Tie-min Zhou

**Affiliations:** School of Educational Science, Shenyang Normal University, Shenyang, 110034 China

**Keywords:** Time-to-collision (TTC), Background information, Speed of motion, Horizontal-vertical illusion

## Abstract

**Background:**

In previous studies, most research on motion perception have been conducted under background-free condition when the stimulus moved in a plane parallel to the observer. In real-life situations, people’s perception of the motion state of objects is usually done under different visual noise. Based on the occlusion paradigm, this study aimed to investigate whether different background information and motion speed affect the trend and accuracy of time-to-collision (TTC) estimation when stimuli move in a plane parallel to the observer.

**Methods:**

Thirty five college students (mean age = 20.94, SD = 2.95, range = 18-28 years) participated in experiment 1, and used a 2 (background orientation: horizontal, vertical) × 3 (motion speed: slow, medium, fast) design to explore the effect of different line segment orientations and motion speed on TTC estimation performance; 36 college students (mean age = 20.81, SD = 2.82, range = 18-28 years) participated in experiment 2, and used a 2 (background dimension: two-dimensional background, three-dimensional background) × 3 (motion speed: slow, medium, fast) design to explore the effect of different background dimensions and motion speed on the performance of TTC estimation. The data were analyzed using SPSS 25.0.

**Results:**

The results revealed that: (1) The TTC was underestimated for the slow speed condition and overestimated for the medium and fast speed conditions. (2) The highest accuracy of TTC estimation was obtained for the fast condition. (3) The TTC were overestimated for the vertical background condition and underestimated for the horizontal background condition. (4) Compared to the two-dimensional background, the TTC was overestimated in the three-dimensional background.

**Conclusions:**

Object motion speed affected the TTC estimation performance, and different background information affected the TTC estimation performance when the object moved in a plane parallel to the observer. Meanwhile, the impact of background orientation and motion speed showed significant interactions.

**Supplementary Information:**

The online version contains supplementary material available at 10.1186/s40359-023-01502-x.

## Introduction

In many situations, estimating when a moving object will reach a given position is a very important skill. For example, to catch a football flying through the air, a player must estimate when the football will reach his feet; a pedestrian decides whether to cross the road immediately or to stop and wait by estimating the speed of a vehicle; a driver regulates the speed of a vehicle in time by estimating the speed of the vehicle in front of him and determining the distance between them. This task of determining when a moving object will reach a given location is known as the time-to-collision (TTC) estimation task [1–3].

In recent years, in some places, zebra crossings have been painted as three-dimensional graphics, using three-dimensional forms of zebra crossings to simulate roadblocks, so that drivers have the optical illusion that they are “speed bumps” highlighting the road, and thus can make timely braking actions [4], and researchers conducted a field study that three-dimensional crosswalks are effective in reducing vehicle speeds [5]. It has been found that an individual’s judgment of an object’s state of motion can be disturbed by background information [6], that scenes with depth perceptual cues can increase an individual’s cognitive load [7]. In vehicle driving, accurate estimation of crash time is an important factor affecting driving safety and driving comfort. Whether depth perceptual cues lead to reduced performance in TTC estimation and whether three-dimensional zebra crossings interfere with drivers’ judgments are questions that will be explored in this study.

### Affecting factors of TTC estimation task

It has been shown that individuals can infer the remaining time for the stimulus to reach the individual from the ratio of the angle at which the motion stimulus is imaged on the retina to the rate of change of this angle when the stimulus approach an observer [8–10]. However, when the stimulus moved in a plane parallel to the observer, there was no visual expansion information about the stimulus itself, and the observer calculated the TTC of the object by effectively estimating the speed as well as the distance of the moving object and using higher-level thinking activities [11–15]. Individuals with higher cognitive ability can realize that both speed and distance information in the situation can influence the TTC estimation, and can effectively integrate speed and distance information in the task to estimate more accurately [6, 13, 16, 17]. Yan and You (2015) found that in the relative arrival time task, compared to the pilot group, the control group was more susceptible to background information, longer reaction times in the tilted target line condition than in the vertical target line, and lower correct judgment rates [6].

### Horizontal-vertical illusion

The horizontal-vertical illusion (HVI) refers to the fact that in an inverted “T” structure consisting of two lines of equal length, the vertical line segment is often perceived as longer than the horizontal one [18, 19]. Künnapas (1955) found that the greatest amount of illusion was generated when the vertical line segment was located in the middle of the horizontal line segment, and as the segmentation position moved toward the ends of the horizontal line segment, the amount of illusion decreased. Therefore, researchers believed that the horizontal-vertical illusion was the result of a combination of overestimation of the vertical line segment and overestimation of the segmentation line [20, 21]. By rotating the inverted “T” figure in the plane, researchers found that the amount of illusion caused by pure segmentation illusion was higher than that of pure vertical-horizontal illusion [20, 22]. Moreover, the illusion effect was most pronounced when the angle between the horizontal and vertical lines was 90°, and increasing or decreasing the “angle” reduced the amount of illusion [23]. Since the vertical line segment divided the horizontal line segment, this phenomenon was also referred to as the segmentation illusion.

In previous TTC estimation task, when the stimulus moved in a plane parallel to the observer, the researchers were more likely to be in a background-free condition [3, 16, 17, 24, 25]. However, an object’s motion in realistic environments contained background changes and visual noise [6], so exploring performance differences in TTC under different background conditions is closer to realistic contexts. This study used the occlusion paradigm, where the moving object was visible during the initial motion and it became invisible after encountering an occluder, and the observer had to imagine that it continued to move to estimate and judge the time for the moving object to reach the specified location [3, 25–27], to explore the effect of different background information on the performance of TTC estimation. Experiment 1 used line segments of different orientations as backgrounds to compare the effects of different backgrounds on the performance of TTC estimation due to the effect of the segmentation illusion by constructing path of movement for motion stimuli with different structural relationships (horizontal or vertical) posed by the line segments of different orientations. Experiment 2 used patterns of different dimensions as backgrounds to compare the effects of different backgrounds on TTC estimation due to the fact that depth perception cues increase an individual’s cognitive load. Meanwhile, since the backgrounds in the experiment 2 by using equal proportionally scaled conventional zebra crossing and three-dimensional zebra crossing, the experimental 2 results were used to explore the reasonableness of the two kinds of zebra crossing, and to provide bases for the improvement and application of zebra crossing. The study selected two performance indicators for analysis: the constant error (CE) (the difference between the response time and the actual time) and the absolute error (AE) (the absolute value of CE); the indicator of CE focused on the tendency and extent to which individuals overestimated or underestimated when performing TTC estimation, while the indicator of AE focused on the accuracy of individual TTC estimation [3].

### Experiment 1

Experiment 1 used a 2 (background orientation: horizontal, vertical) × 3 (motion speed: slow 100pixel/s, medium 200pixel/s, fast 300pixel/s) two-factor within-subject experimental design. The size ratio of speed we set in the experiment was consistent with the study of Tian Yu et al. (2018), in whose study the size ratio of slow, medium, and fast speeds was 1:2:3 [25].

## Materials and methods

### Participants

The sample size was estimated prior to the implementation of the experiment using the G*power 3.1.9.7 software with the settings of *f* = 0.25, α = 0.05, 1-β = 0.80, and the sample size resulting from the calculations was 29 [28]. In order to prevent invalidity of the sample data, undergraduate and graduate students from a provincial normal university were selected as the participants of the study, which consisted of a total of 35 students (mean age 20.94 ± 2.95 years old). The participants were all right-handed, with normal vision or corrected vision and no color blindness or color weakness. Each participant volunteered to participate in the experiment. They had not participated in such experiments before. The appropriate fee was given at the end of the experiment. All participants provided written informed consent after study procedures were explained to them thoroughly, and were informed that they were free to withdraw from the study at any time during the test.

### Apparatus

The experimental program was written using E-prime 3.0.3.9 software. The experimental stimuli were presented on a 23.8-inch Redmi 1A monitor with a display size of 539.2 × 419.5 mm, a screen resolution of 1920 × 1080 (horizontal by vertical), and a refresh rate of 60 Hz. The distance between the participants and the screen was approximately 60 cm during the experiment. The time of each trial presented was determined according to the speed of the green ball motion, which was 6200 ms (fast), 7550 ms (medium), and 11600 ms (slow).

### Experimental materials

**Fig. 1:**
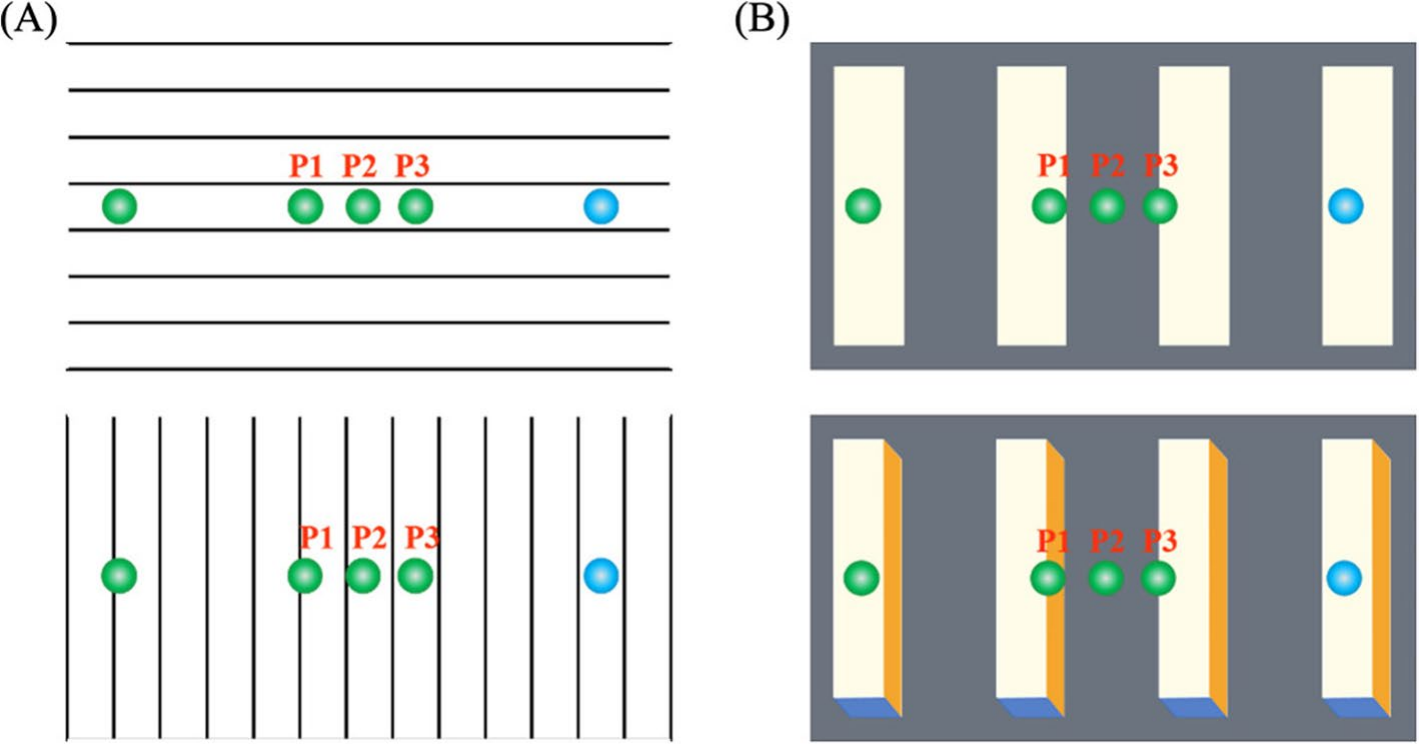
**A** The example of stimuli used in Experiment 1; **B** The example of stimuli used in Experiment 2

Eighteen videos in avi format produced by After Effect (version 2020) were used for the experimental materials. The video size was1000 × 540 pixels (length by height), the width of each line was about 2.6 pixels (0.1°), and the distance between every two lines was about 74.1 pixels (1.9°). The interval distance between line segments was the same under different orientations The diameter size of the small balls located on the left and right sides of the video both were 60 pixels (1.4°), including the green ball (RGB: 30, 180, 80) on the left side and the blue ball (RGB: 10, 160, 240) on the right side. The distance between the green and blue balls is 810pixels (18.9°). 500 ms after the video plays, the green ball moves to the blue ball horizontally to the right in a uniform linear motion. The motion speed of the green ball is 100pixel/s (slow), 200pixel/s (medium), and 300pixel/s (fast) respectively. The green ball was masked to the specified position. To prevent subjects from forming memory and practice effects that would interfere with the experimental results, the masking locations were divided into three levels, P_1_: 370pixels, P_2_: 470pixels, and P_3_: 560pixels, respectively (see Fig. 1A).

### Experimental procedure

The experiment was conducted in a psychology laboratory, where the room was kept quiet, with appropriate light and temperature, and maintained constant to exclude the interference of additional variables to the experiment.

Participants were informed before the start of the experiments. A background picture consisting of horizontal line segments or vertical line segments (Experiment 1) or a two-dimensional or three-dimensional background picture (Experiment 2) was presented in the experiments. For the experiments, on the left side of the picture there is a green ball and on the right side there is a blue ball. The green ball will move at a constant speed in a straight line toward the blue ball, in which the green ball will disappear after a certain distance. At this time, the participants are asked to imagine that it will continue to move at the same speed, and when the participants feel that the green ball and the blue ball completely coincide, please press the "b" key. After making sure that the participants understand the presentation of the experiment above, they are asked to do the experiment practice. When the participant is familiar with the experiment procedure, they will begin the formal experiment.

**Fig. 2:**
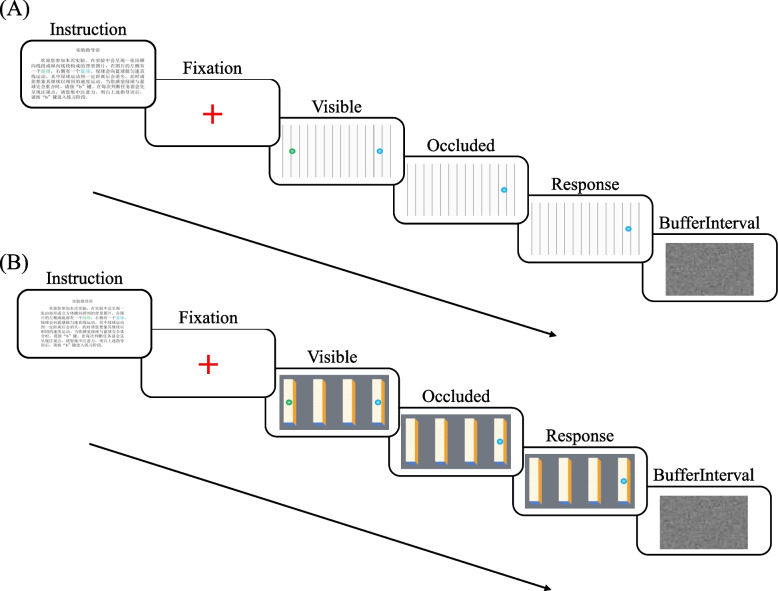
**A** Experimental procedure of Experiment 1; **B** Experimental procedure of Experiment 2

They were first given a practice experiment with 18 trials. After they were familiar with the experimental procedure, they entered the formal experiment. The formal experiment consisted of 5 blocks of 90 trials, each block containing 18 trials. Each trial was presented randomly during the experiment. For the Experimental procedure of Experiment 1, see Fig. 2A.

### Data analysis method

The raw data were preprocessed, and the absolute values of the 15 CE for each participant in each combination of motion speed and background orientation were used as the data unit, and the data whose values were outside the range of “mean ± 3 standard deviations” were excluded, and a total of 18 data were excluded [3, 25]. The data excluded according to the above criteria accounted for 0.57% of the total data.

The constant error and the absolute error of the participants’ TTC estimation were used as data indicators to measure the experimental results. The experimental results were analyzed using SPSS 25.0.

## Results

### Descriptive statistical analysis of the constant error

For each condition formed by the combination of the motion speed and the background orientation, a descriptive statistical analysis of the constant error was performed. The results of the preliminary processing of the data were shown in Table 1.

**Table 1:** Descriptive statistics of the constant error of experiment 1 (*M* ± *SD*)(ms)

	*M*	*SD*
Horizontal-slow	-660.41	534.33
Horizontal-medium	169.25	432.77
Horizontal-fast	212.58	354.29
Vertical-slow	-407.85	488.85
Vertical-medium	341.81	425.71
Vertical-fast	270.92	351.60

### Analysis of variance for the constant error

**Fig. 3:**
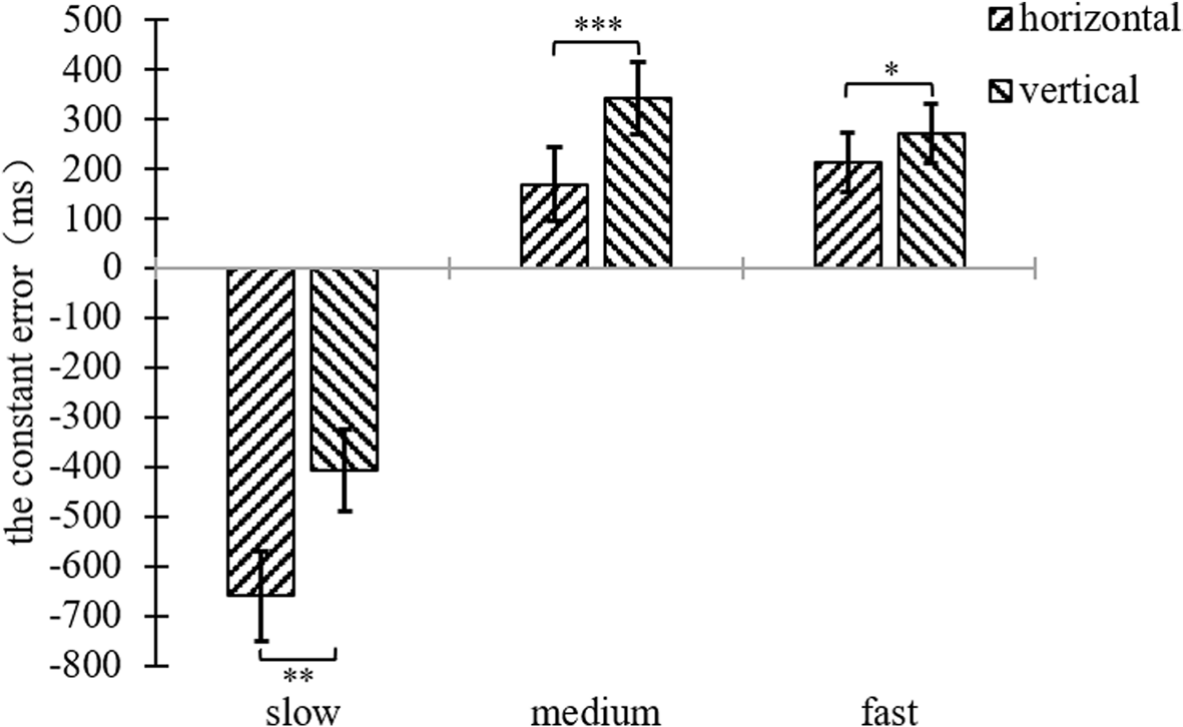
Plot of repeated measurement variance results for the constant error of experiment 1

A two-factor repeated measures ANOVA was performed on the constant error of the participant’s TTC of 2 (background orientation: horizontal, vertical) × 3 (motion speed: slow 100pixel/s, medium 200pixel/s, fast 300pixel/s), and the results were shown in Fig. 3, and Figure S1.

The results showed that the main effect of motion speed was significant, (*F*(2,68) = 124.99, *p* < 0.001, η^2^ = 0.786), and post hoc tests revealed that the constant error in the slow speed condition differed significantly from both the medium and fast conditions (*p* < 0.001), while the difference between the medium and fast conditions was not significant for both (*p* = 0.665). This was demonstrated by the fact that participants tended to underestimate the TTC in the slow speed condition, while they tended to overestimate the TTC in the medium and fast speed conditions.

The main effect of background orientation was significant, (*F*(1,34) = 19.60, *p* < 0.001, η^2^ = 0.366), and the constant error in the horizontal line segment background condition differed significantly from that in the vertical line segment background condition. This was demonstrated by the fact that participants tended to underestimate the TTC in the horizontal line segment background condition, whereas they tended to overestimate the TTC in the vertical line segment background condition.

The interaction between motion speed and background orientation was significant, (*F*(2,68) = 7.07, *p* = 0.002, η^2^ = 0.172). In terms of motion speed, a paired samples t-test for both background orientations was conducted in the slow speed condition, horizontal-vertical (*t*(34) = -3.74, *p* = 0.001). A paired samples t-test for both background orientations was conducted in the medium speed condition, horizontal-vertical (*t*(34) = -4.65, *p* < 0.001). A paired samples t-test for both background orientations was conducted in the fast speed condition, horizontal-vertical (*t*(34) = -2.15, *p* = 0.039). The constant error was significant in the slow, medium, and fast speed conditions with different background orientations. This was evidenced by the tendency of participants to underestimate TTC in the horizontal line segment background condition and overestimate TTC in the vertical line segment background condition, regardless of speed conditions.

**Fig. 4:**
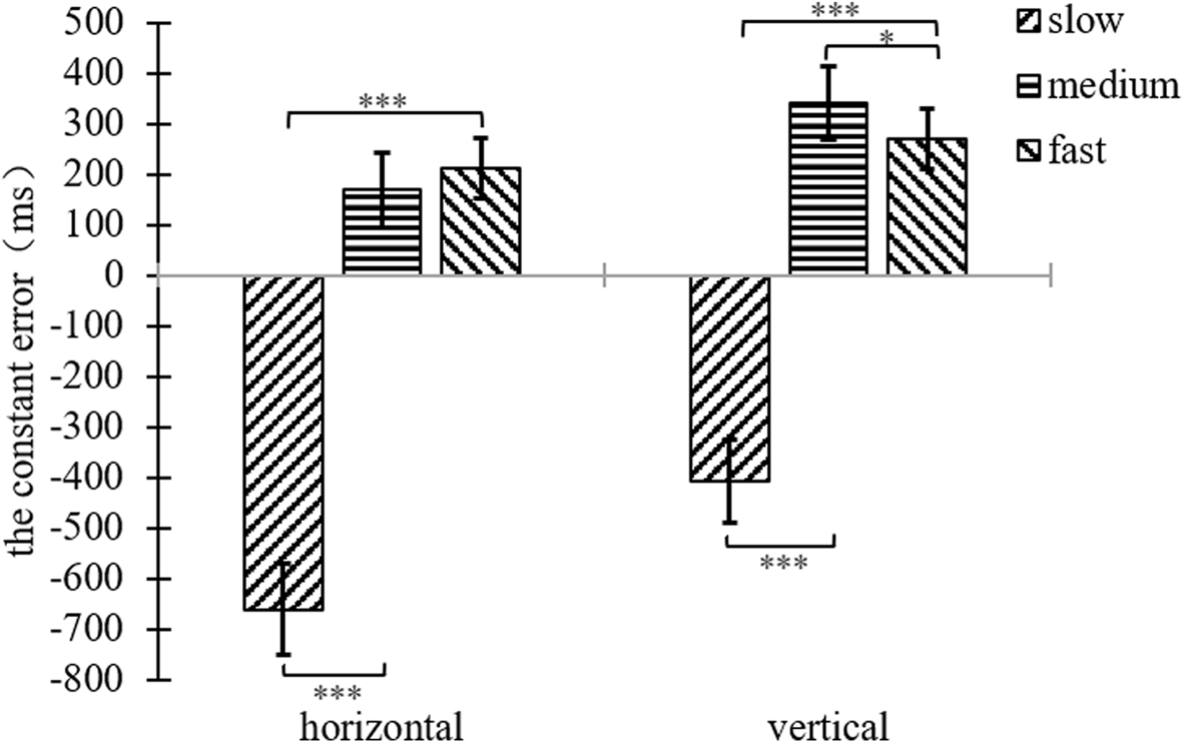
Plot of the result of the interaction of speed and background orientation for the constant error of experiment 1

In terms of background orientation, the three motion speeds differed significantly in the horizontal line segment background condition (*F*(2,33) = 74.34, *p* < 0.001, η^2^ = 0.818), and paired samples t-tests were performed for the three motion speeds, slow-medium (*t*(34) = -12.28, *p* < 0.001), and slow-fast (*t*(34) = -11.36, *p* < 0.001), with non-significant differences between the medium and fast conditions (*p* = 0.274). In the horizontal line segment background condition specifically, participants tended to underestimate TTC in the slow speed condition and overestimate TTC in the medium speed and fast conditions; In the vertical line segment background condition, the three motion speeds differed significantly (*F*(2,33) = 58.11, *p* < 0.001, η^2^ = 0.779), and paired samples t-tests were performed for the three motion speeds, slow-medium (*t*(34) = -10.85, *p* < 0.001), slow-fast (*t*(34) = -9.00, *p* < 0.001), and medium-fast (*t*(34) = 2.04, *p* = 0.049) (see Fig. 4). In the vertical line segment background condition specifically, participants in the slow speed condition tended to underestimate TTC, participants in the medium and fast speed conditions tended to overestimate TTC and participants in the medium speed condition produced greater overestimation.

### Descriptive statistical analysis of the absolute error

For each condition formed by the combination of the motion speed and the background orientation, the participants performed a descriptive statistical analysis of the absolute error. The results of the preliminary processing of the data were shown in Table 2.

**Table 2:** Descriptive statistics of the absolute error of experiment 1 (*M* ± *SD*)(ms)

	*M*	*SD*
Horizontal-slow	737.96	416.98
Horizontal-medium	348.29	303.16
Horizontal-fast	320.29	257.76
Vertical-slow	514.39	371.24
Vertical-medium	442.12	316.67
Vertical-fast	347.85	273.17

### Analysis of variance for the absolute error

**Fig. 5:**
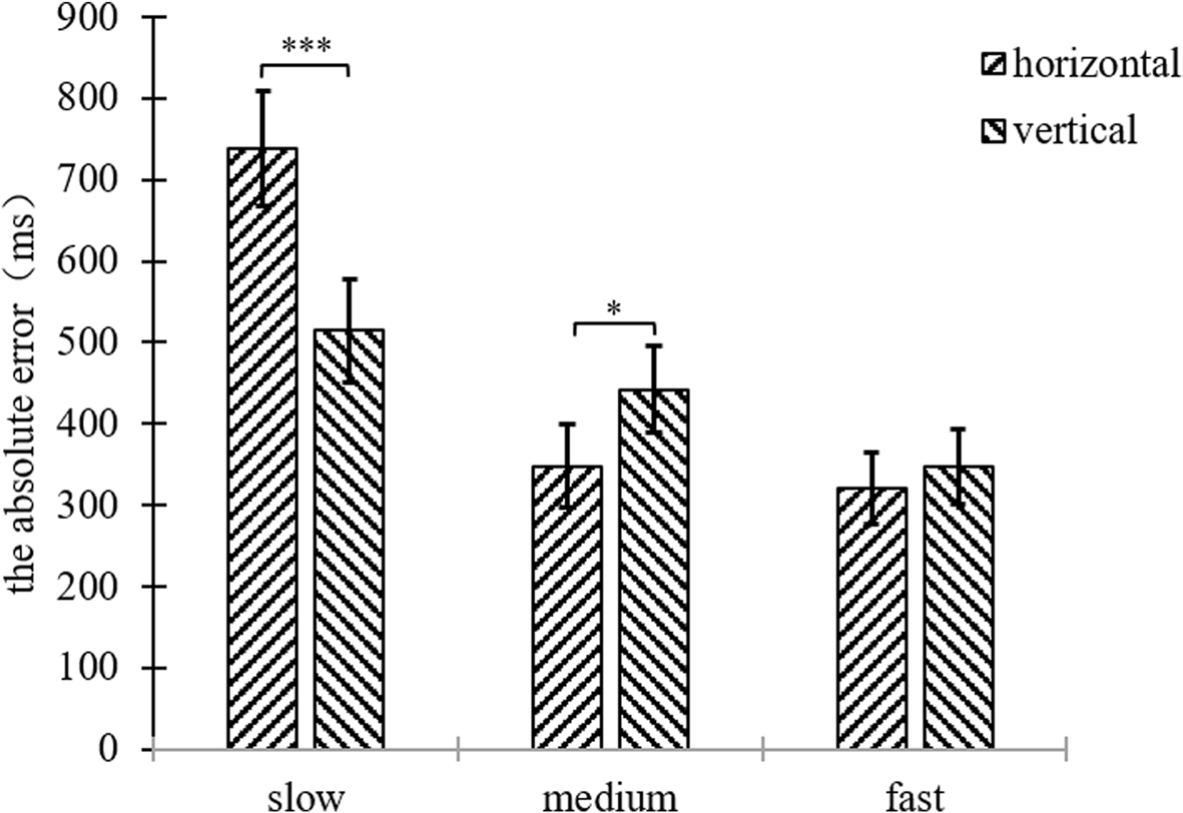
Plot of repeated measurement variance results for the absolute error of experiment 1

A two-factor repeated measures ANOVA was performed on the absolute error of the participant’s TTC of 2 (background orientation: horizontal, vertical) × 3 (motion speed: slow 100pixel/s, medium 200pixel/s, fast 300pixel/s), and the results were shown in Fig. 5, and Figure S2.

The results showed that the main effect of motion speed was significant, (*F*(2,68) = 10.27, *p* < 0.001, η^2^ = 0.232), and the post hoc test showed that the difference in the absolute error were two-by-two significant in the slow-medium (*p* = 0.010), medium-fast (*p* = 0.033), and slow-fast (*p* = 0.001) conditions. Specifically, the absolute error decreased as the speed increased and the more accurately the participants estimated the TTC.

The absolute error was not significant differences between the different background orientations, (*F*(1,34) = 2.31, *p* = 0.138).

The interaction between motion speed and background orientation was significant, (*F*(2,68) = 15.18, *p* < 0.001, η^2^ = 0.309). In terms of motion speed, a paired samples t-test for both background orientations was conducted in the slow speed condition, horizontal-vertical (*t*(34) = 3.99, *p* < 0.001). In the slow speed condition specifically, participants in the vertical line segment background condition estimated TTC more accurately. A paired samples t-test for the two background orientations was conducted in the medium speed condition, horizontal-vertical (*t*(34) = -2.54, *p* = 0.016). In the medium speed condition, participants in the horizontal line segment background condition estimated the TTC more accurately. The difference between the two background orientations in the fast condition was not significant (*p* = 0.303).

**Fig. 6:**
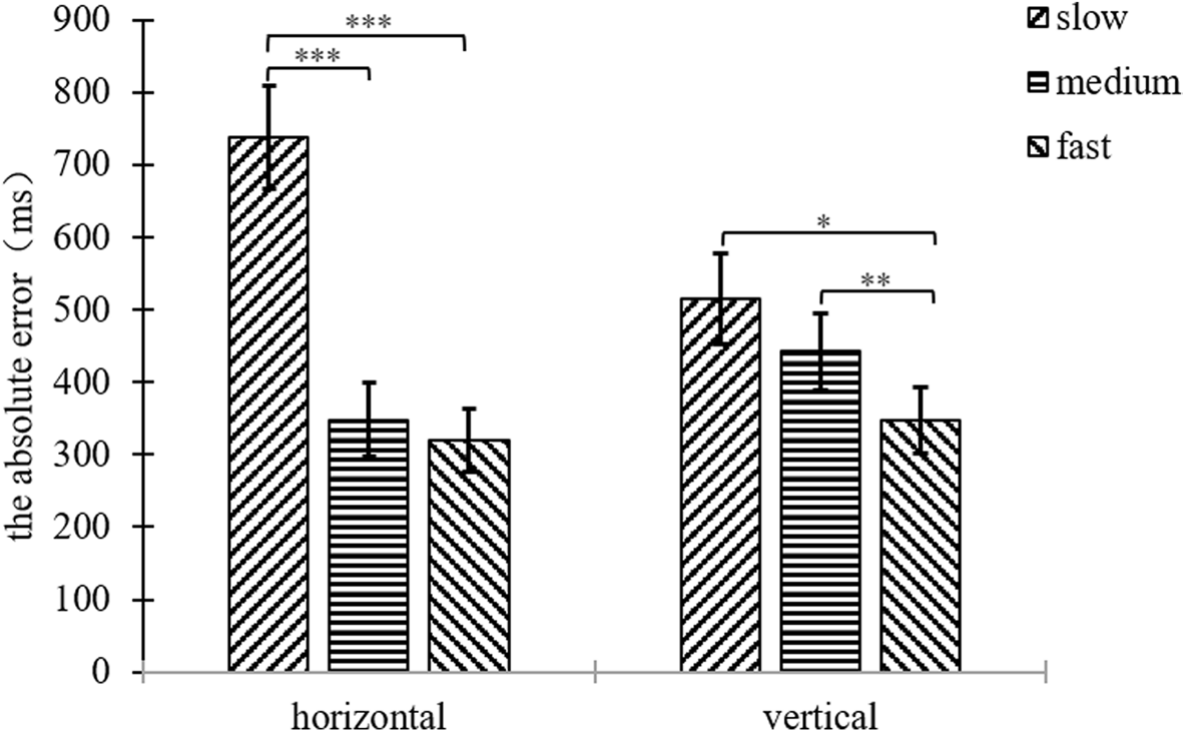
Plot of the result of the interaction of speed and background orientation for the absolute error of experiment 1

In terms of background orientation, the three motion speeds differed significantly in the horizontal line segment background condition (*F*(2,33) = 12.22, *p* < 0.001, η^2^ = 0.426). Paired samples t-tests were performed for the three motion speeds, slow-medium speed (*t*(34) = 4.11, *p* < 0.001), and slow-fast (*t*(34) = 4.88, *p* < 0.001), with no significant differences between the medium and fast speed conditions (*p* = 0.429). In the horizontal line segment background condition specifically, participants’ estimation of TTC were more accurate in the medium and fast speed conditions compared to the slow speed condition; In the vertical line segment background condition, the three motion speeds differed significantly (*F*(2,33) = 7.55, *p* = 0.002, η^2^ = 0.314), and paired-sample t-tests were performed for the three motion speeds, slow-fast (*t*(34) = 2.04, *p* = 0.049), medium-fast (*t*(34) = 3.10, *p* = 0.004). The differences between the slow and medium speed conditions were not significant (*p* = 0.429) (see Fig. 6). In the vertical line segment background condition, implying that participants in the fast condition estimated the TTC more accurately compared to the slow and medium speed conditions.

## Discussion

### Effect of motion speed on the performance of TTC estimation

The present study found that in the relatively slow speed condition, participants tended to underestimate the TTC of the stimulus and perform the keystroke response earlier, while in the medium and fast speed conditions, participants tended to overestimate the TTC of the stimulus and delay the keystroke response. This was consistent with the findings of previous studies [11]. When the motion speed of the stimulus increased, individuals tended to extend the estimation of time; in contrast, when the motion speed of the stimulus decreased, the phenomenon of early response emerged [29].

The more accurate the participants’ TTC estimation with increasing motion speed was consistent with previous studies, which showed a positive correlation between motion speed and the accuracy of TTC estimation [30, 31]. It has been shown that in a TTC estimation task, when the motion distance was certain, the longer the motion time was, the more susceptible participants were to interference from other factors. This led to a decrease in estimation performance [32, 33].

### Effect of background orientation on the performance of TTC estimation

DeLucia (2013) found that information sources that do not provide accurate information about TTC can influence TTC judgments [34]. The present study found that participants tended to underestimate the TTC in the horizontal line segment background condition, while they tended to overestimate the TTC in the vertical line segment background condition. Since the movement path of the motion stimulus in the present study was related to the line segments as vertical and equally divided by constructing a vertical context, which was an important inducing factor in horizontal-vertical illusion [21, 22], participants overestimated the TTC of the motion stimulus in the vertical context where the perceptual motion path was extended.

Although participants did not show differences in the accuracy of TTC estimation in different backgrounds, the mean of the absolute error was larger in different backgrounds, combined with the effect of background orientation on the constant error, suggested that backgrounds can affect the TTC estimation task when the moving stimulus was moving in a plane parallel to the observer [6].

### Interaction effect of motion speed and background orientation

In the vertical line segment background, the medium speed condition showed a greater degree of overestimation compared to the fast condition. It has been shown that participants integrated information about relative distance and relative speed in TTC estimation task and would over-rely on distance information in their judgments [6, 16]. Due to the presence of the segmentation illusion, the background of the vertical line segment affected the participants’ perception of distance information. In the medium speed condition, when the motion stimulus was occluded, the motion representation of the stimulus might be more influenced by the background orientation, so exhibiting a greater degree of overestimation.

In the slow speed condition, participants estimated the TTC more accurately in the vertical line segment background; while in the medium speed condition, participants estimated the TTC more accurately in the horizontal line segment background. After the experiments were completed, it was found through participants’ subjective reports that subjects generally responded to the TTC estimation by estimating the stimulus elapsed time between line segments when the stimulus was occluded in the vertical line segment background, even though the line segments interval was larger than the motion stimulus itself. It has been suggested that distance representation was a fundamental factor influencing motion time judgments and is more likely to be preferentially used as a cue in judgments [6]. In the slow speed condition, participants may have allocated more attention to the background and estimated the time of the stimulus passing through each two vertical line segments during the visual phase, thus completing the TTC estimation task by estimating the time of the stimulus passing through each two vertical line segments during the occlusion phase. In contrast, without this reference frame in the horizontal line segment background, the estimation accuracy decreased. Compared to the slow speed condition, the increased motion speed of the stimulus in the medium speed condition might affect participants’ cognitive processing of the stimulus motion representation due to the background construction of the vertical line segment, which perceptually tended to prolong the motion distance of the stimulus, and the accuracy of TTC estimation in the vertical background decreased.

### Experiment 2

Experiment 2 used a 2 (background dimension: two-dimensional background, three-dimensional background) × 3 (motion speed: slow 100pixel/s, medium 200pixel/s, fast 300pixel/s) two-factor within-subject experimental design. The size ratio of speed we set in the experiment was consistent with the study of Tian Yu et al. (2018), in whose study the size ratio of slow, medium, and fast speeds was 1:2:3 [25].

## Materials and methods

### Participants

The number of participants was calculated and selected in the same way as in Experiment 1. Thirty-six undergraduate and graduate students (mean age 20.81 ± 2.82 years old) were recruited from a provincial normal university. The participants were all right-handed, with normal vision or corrected vision and no color blindness or color weakness. Each participant volunteered to participate in the experiment, and none had participated in such experiments before. The appropriate fee was given at the end of the experiment. All participants provided written informed consent after study procedures were explained to them thoroughly, and were informed that they were free to withdraw from the study at any time during the test.

### Apparatus

Same as experiment 1.

### Experimental materials

A total of eighteen videos in avi format were used for this experimental material, and the video materials were also produced using After Effect (version 2020). The video presentation sizes were all 1000 × 540pixels (length by height), and the video background color of materials were gray (RGB: 100, 110, 120). In the two-dimensional background, each rectangle was approximately 422pixels × 115pixels (11.7° × 2.8°), the color was white (RGB: 255, 252, 233), and the rectangles were spaced approximately 154pixels (3.7°) apart. In the three-dimensional background, each cuboid was approximately 392pixels (length) × 86pixels (width) × 31pixels (height) (10.9° × 2° × 0.8°), the front color was white (RGB: 255, 252, 233), the side color was yellow (RGB: 255, 153, 0), and the bottom color was blue (RGB: 68, 114, 196). The cuboids’ intervals were about 154pixels (3.7°) (see Fig. 1 (B)). All other specific parameters, as well as the video presentation, were the same as in experiment 1.

### Experimental procedure

The Experimental procedure of Experiment 2. see Fig. 2 (B).

All the other experimental procedure were the same as experiment 1.

### Data analysis method

The raw data were preprocessed, and the absolute values of the 15 CE for each participant in each combination of motion speed and background dimension were used as data units, and data with values outside the range of “mean ± 3 standard deviations” were excluded, resulting in a total of 15 data being excluded [3, 25]. The data excluded according to the above criteria accounted for 0.46% of the total data.

The constant error and the absolute error of the participants’ TTC estimation were used as data indicators to measure the experimental results. The experimental results were analyzed using SPSS 25.0.

## Results

### Descriptive statistical analysis of the constant error

For each condition formed by the combination of the motion speed and the background dimension, the participants performed a descriptive statistical analysis of the constant error, and the results of the preliminary processing of the data were shown in Table 3.

**Table 3:** Descriptive statistics of the constant error of experiment 2 (*M* ± *SD*)(ms)

	*M*	*SD*
Two-dimensional-slow	-419.44	531.20
Two-dimensional-medium	296.24	433.30
Two-dimensional-fast	303.74	309.03
Three-dimensional-slow	-341.60	608.80
Three-dimensional-medium	362.19	458.56
Three-dimensional-fast	356.58	320.42

### Analysis of variance for the constant error

**Fig. 7:**
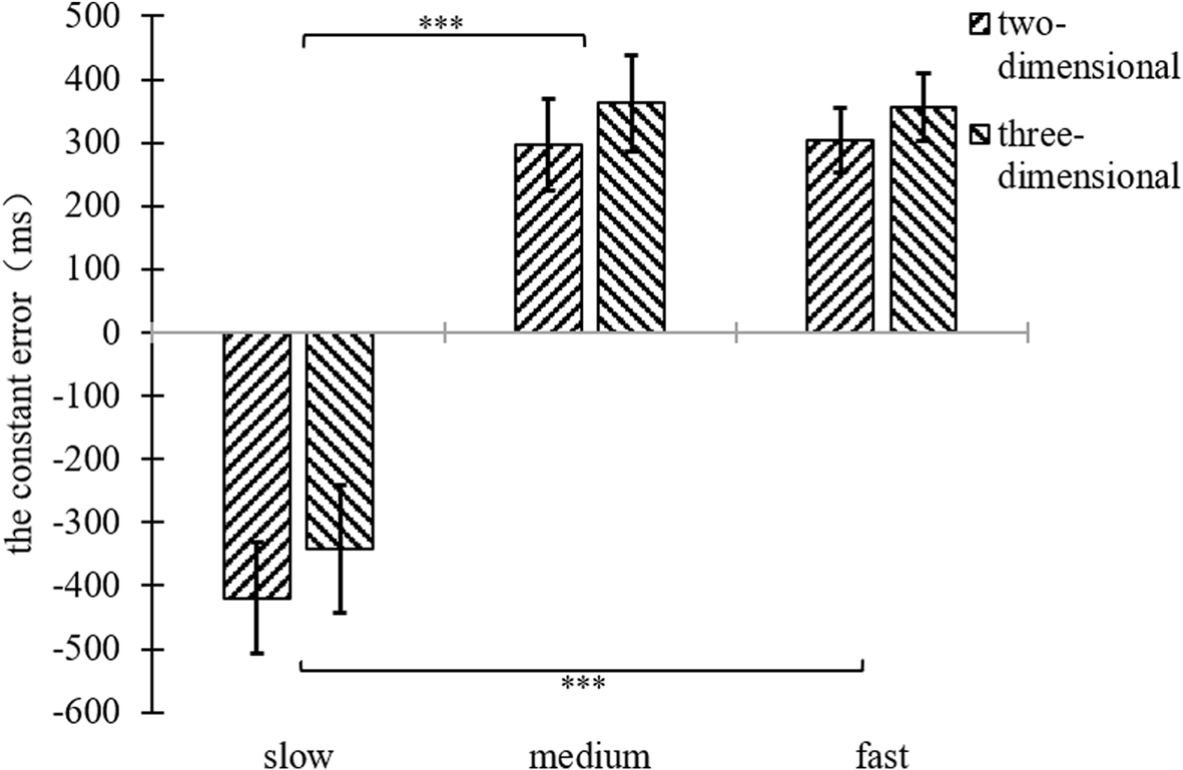
Plot of repeated measurement variance results for the constant error of experiment 2

A two-factor repeated measures ANOVA was performed on the constant error of the participant’s TTC of 2 (background dimension: two-dimensional, three-dimensional) × 3 (motion speed: slow 100pixel/s, medium 200pixel/s, fast 300pixel/s). The results are shown in Fig. 7, and Figure S3.

The results showed that the main effect of motion speed was significant, (*F*(2,70) = 69.18, *p* < 0.001, η^2^ = 0.664), and post hoc tests revealed that the constant error in the slow speed condition differed significantly from both the medium and fast conditions (*p* < 0.001), while the difference between the medium and fast conditions was not significant (*p* = 0.981). This was demonstrated by the fact that participants tended to underestimate the TTC in the slow speed condition, while they tended to overestimate the TTC in the medium and fast speed conditions.

The main effect of the background dimension was significant, (*F*(1,35) = 8.51, *p* = 0.006, η^2^ = 0.196), and the constant error in the two-dimensional background condition was significantly different from that in the three-dimensional background condition. This was shown by the tendency to overestimate the TTC in the three-dimensional background condition compared to the two-dimensional background condition.

The interaction between motion speed and background dimension was not significant, (*F*(2,70) = 0.169, *p* = 0.845).

### Descriptive statistical analysis of the absolute error

For each condition formed by the combination of the motion speed and the background dimension, the participants performed descriptive statistical analysis of the absolute error, and the results of the preliminary processing of the data were shown in Table 4.

**Table 4:** Descriptive statistics of the absolute error of experiment 2 (*M* ± *SD*)(ms)

	*M*	*SD*
Two-dimensional-slow	526.96	421.33
Two-dimensional-medium	381.95	357.72
Two-dimensional-fast	309.56	303.03
Three-dimensional-slow	531.59	447.22
Three-dimensional-medium	404.29	420.81
Three-dimensional-fast	360.15	316.29

### Analysis of variance for the absolute error

**Fig. 8:**
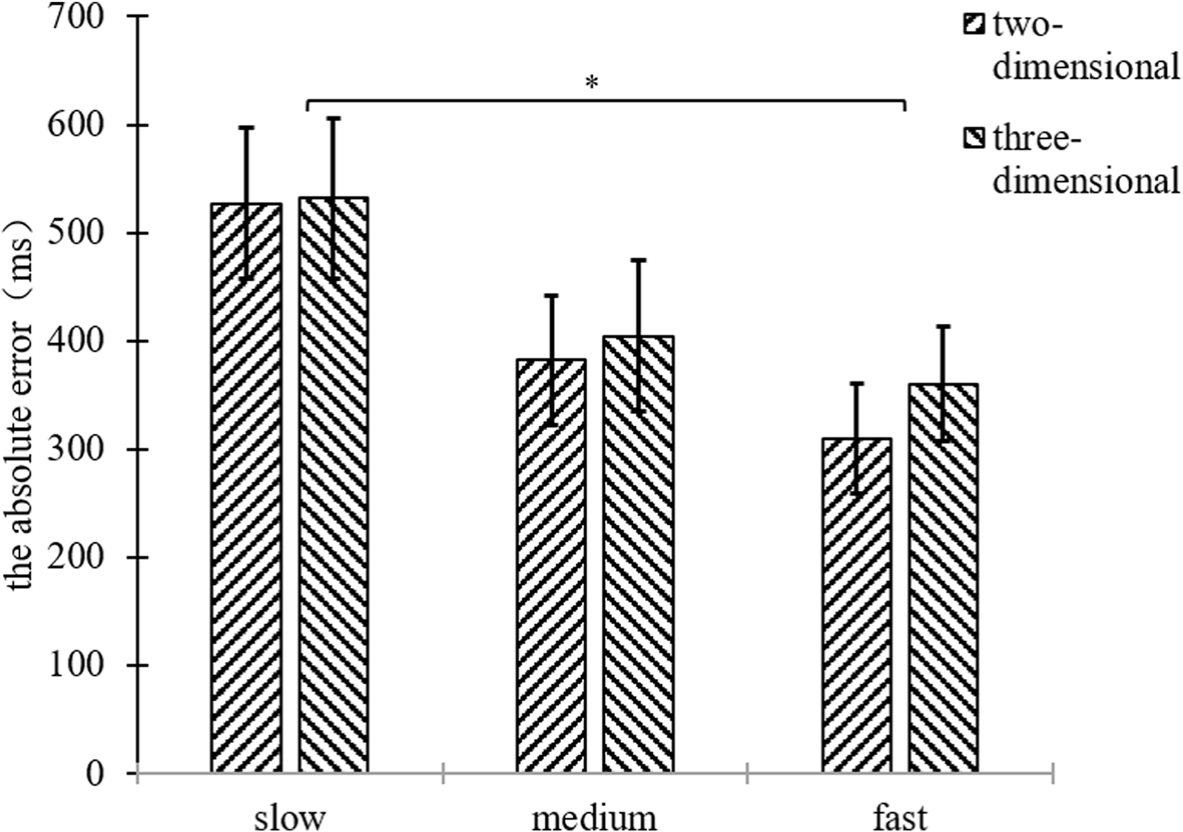
Plot of repeated measurement variance results for the absolute error of experiment 2

A two-factor repeated measures ANOVA was performed on the absolute error of the participant’s TTC of 2 (background dimension: two-dimensional, three-dimensional) × 3 (motion speed: slow 100pixel/s, medium 200pixel/s, fast 300pixel/s), and the results were shown in Fig. 8, and Figure S4.

The results showed that the main effect of motion speed was significant, (*F*(2,70) = 3.24, *p* = 0.045, η^2^ = 0.085). Post hoc tests revealed that the differences between the slow and fast conditions was significant (*p* = 0.025), the marginal differences between the medium and fast conditions was significant (*p* = 0.075), and the differences between the slow and medium conditions was not significant (*p* = 0.194). This was demonstrated by the fact that the absolute error was smallest in the fast condition compared to the slow and medium conditions, and the participants’ estimation of the TTC was accurate. Moreover, the absolute error was not significant differences between the different background dimension, (*F*(1,35) = 1.30,* p* = 0.262). The interaction between motion speed and background dimension was not significant, (*F*(2,70) = 0.588, *p* = 0.558).

## Discussion

The study found that different background dimensions had difference in the constant error, although the accuracy of the TTC in the two background dimensions did not show statistical differences. However, compared to the two-dimensional background, the three-dimensional background condition tended to overestimate the TTC more and tended to delay the keystroke response. It has been shown that in condition with stereo vision, the amount of extraneous information contained also increased significantly due to the presence of depth perceptual cues, and the perception of spatial capacity and the structural relationships in spatial proximity were more complex, making the information capacity of the scene showed a geometric increase, thus creating information overload [35]. It was found that the difference in search efficiency on repetitive scenes and novel scenes differed in different dimensional contexts, and the difference in search efficiency exhibited by the two scenes was smaller in a two-dimensional context than in a three-dimensional scene, and the difference in search efficiency was greater [7]. Researchers believed that the possible reason for this difference is the complexity of three-dimensional spatial scenes, which made depth information on cognitive processing to a certain interference effect. In the present study, compared to the two-dimensional background, the three-dimensional background might have taken up more processing resources and increased the cognitive load on visual attention, leaving the motion stimuli incomplete for cognitive processing [36, 37], resulting in greater deviation in the TTC estimation. For fast motion stimuli, participants may focus more on the motion stimuli itself and be less disturbed by the background due to the increased motion speed and reduced motion time, and thus show higher accuracy compared to the medium and slow speed conditions [3, 32].

### General discussion

In this study, TTC were underestimated in the slow speed condition and overestimated in the medium and fast speed conditions. TTC estimation was more accurately as speed increased in experiment 1. Moreover, TTC estimation was more accurately in the fast condition.

In Experiment 1 in the vertical line segment background condition, participants tended to overestimate TTC; and in the vertical line segment background condition, participants tended to produce greater overestimation of TTC in the medium speed condition. In the slow condition, participants estimated TTC more accurately in the vertical line segment background condition; and in the medium speed condition, participants estimated TTC more accurately in the horizontal line segment background condition. In the fast condition, there was no significant difference in the accuracy of the TTC estimation between the two background conditions. In Experiment 2 in the three-dimensional background condition, participants tended to overestimate TTC.

### Effect of motion speed on the performance of TTC estimation

The present study found a significant effect of motion speed on the performance of TTC estimation, suggesting that motion speed is an important stimulus characteristic factor affecting TTC estimation [25, 30, 31]. Irrespective of whether the backgrounds were horizontal or vertical, two-dimensional or three-dimensional, the slow speed condition underestimated the TTC of the stimuli, while the medium and fast speed conditions tended to overestimate the TTC of the stimuli. This was consistent with the results of previous studies [25]. Unlike the results of Experiment 1, the accuracy of TTC estimation did not improve with increasing speed in Experiment 2 and was more accurately estimated in the fast condition, while the difference between the slow and medium speed conditions was not significant. It has been shown that in a TTC estimation task, when the motion distance was certain, the longer the motion time was, the more susceptible participants were to interference from other factors [32, 33]. Compared to the line segment background, the graphical background may take up more cognitive resources, and the slow and medium speed conditions are more susceptible to interference from background factors due to prolong motion times compared to the fast condition, which affects the performance of TTC estimation.

### Effect of background information on the performance of TTC estimation

It was found that background information exerted a significant influence on the performance of TTC estimation [6]. This was demonstrated by the tendency to show overestimation of TTC in the vertical line segment condition, suggesting that background orientation influenced the cognitive processing of individuals. Participants in the slow speed condition might make TTC estimation with the help of the interval distance provided by the vertical line segment, and tended to shorten the time estimation in the horizontal line segment due to the slow speed. The motion speed of the stimulus increased in the medium speed condition, and the vertical line segment might interfere with the motion representation of the stimulus with reduced accuracy due to the segmentation illusion. Participants in the fast condition might focus more attention on the stimulus itself and so be less affected by interference from the background. A greater tendency to overestimate TTC arose in the three-dimensional background with depth perception. In the background of three-dimensional structures, the TTC estimation task was somewhat disturbed by the influence of depth perceptual cues, which complicated the processing of information about spatial places and thus consumed more cognitive resources, resulting in a larger estimation constant error in the three-dimensional background than in the two-dimensional background [7].

The findings supported the information processing theory of motion perception. In the present study, the moving stimulus made horizontal uniform linear motion in a plane parallel to the observer, the absence of expansion information of the motion stimulus on the retina, and the experimental results suggested that motion speed and background information could have an impact on TTC estimation performance [13, 14, 17].

As a new form of road traffic marking, the three-dimensional crosswalk could indeed attract drivers' attention and alertness and bright better deceleration effect [4, 5], but it might also cause some drivers to be highly nervous and thus made emergency braking behavior, which might lead to serious traffic accidents [38–40]. It might also cause cognitive and recognition difficulties and distractions, which could also lead to safety hazards when pedestrians or drivers take them for granted [5, 41]. In the present study, from the perspective of motion perception, by comparing the performance of TTC estimation in different dimensional backgrounds, it was concluded that although the accuracy of TTC estimation did not show differences in different dimensional backgrounds, it showed a greater tendency of overestimation in the background of three-dimensional. In the real road traffic environment, three-dimensional crosswalks may produce larger cognitive deviation due to depth perception, such as drivers who originally need to slow down or even stop to wait for pedestrians and non-motorized vehicles to pass the lane they are in, but may fail to make timely braking behavior or emergency braking due to larger visual deviation, which may easily cause more serious traffic accidents. Therefore, in summary, the present study concluded that compared to conventional crosswalks, three-dimensional crosswalks might increase the visual deviation of drivers and increase the probability of traffic accidents.

### Limitations

This study was conducted at the behavioral level on TTC estimation in different background information, and the experimental data were not collected with neural indicators or explored and analyzed in terms of neural mechanisms, so no discussion of this part of the neural mechanisms was added in the paper.

## Conclusions

In summary, different motion speeds affected TTC estimation performance. Moreover, different backgrounds affected TTC estimation performance when the object is moving in a plane parallel to the observer. Further, background orientations affected TTC estimation performance differently for different speeds of motion.

### Supplementary Information

**Additional file 1: Figure S1. **Plot of motion speed of the constant error of experiment 1 of the differences between conditions (by subtracting the individual values in the 'slow' condition from the other conditions). **Figure S2.** Plot of motion speed of the absolute error of experiment 1 of the differences between conditions (by subtracting the individual values in the 'slow' condition from the other conditions). **Figure S3.** Plot of motion speed of the constant error of experiment 2 of the differences between conditions (by subtracting the individual values in the 'slow' condition from the other conditions). **Figure S4.** Plot of motion speed of the absolute error of experiment 2 of the differences between conditions (by subtracting the individual values in the 'slow' condition from the other conditions).

## Data Availability

The data that support the findings of this study are available on request from the corresponding author.
