# Supplementary material for: The effect of background information and motion speed on the performance of TTC estimation
Source: BMC Psychol. 2024 Jan 5;12:17. doi: 10.1186/s40359-023-01502-x (PMC10770939; doi:10.1186/s40359-023-01502-x)
Supplement: Supplementary file 1 — Additional file 1: Figure S1. Plot of motion speed of the constant error of experiment 1 of the differences between conditions (by subtracting the individual values in the 'slow' condition from the other conditions). Figure S2. Plot of motion speed of the absolute error of experiment 1 of the differences between conditions (by subtracting the individual values in the 'slow' condition from the other conditions). Figure S3. Plot of motion speed of the constant error of experiment 2 of the differences between conditions (by subtracting the individual values in the 'slow' condition from the other conditions). Figure S4. Plot of motion speed of the absolute error of experiment 2 of the differences between conditions (by subtracting the individual values in the 'slow' condition from the other conditions). [file 40359_2023_1502_MOESM1_ESM.docx]

*Supplementary materials*

***Figure S1.*** Plot of motion speed of the constant error of experiment 1 of the differences between conditions (by subtracting the individual values in the 'slow' condition from the other conditions).

***Figure S2.*** Plot of motion speed of the absolute error of experiment 1 of the differences between conditions (by subtracting the individual values in the 'slow' condition from the other conditions).

***Figure S3.*** Plot of motion speed of the constant error of experiment 2 of the differences between conditions (by subtracting the individual values in the 'slow' condition from the other conditions).

***Figure S4.*** Plot of motion speed of the absolute error of experiment 2 of the differences between conditions (by subtracting the individual values in the 'slow' condition from the other conditions).
